# Miniature Manipulator Design and Cartesian Control for Minimally Invasive Transluminal Endoscopic Surgery

**DOI:** 10.3390/mi13122171

**Published:** 2022-12-08

**Authors:** Yanqiang Lei, Yibin Li, Xingyao Zhang, Gang Zhang, Fuxin Du

**Affiliations:** 1 School of Control Science and Engineering, Shandong University, Jinan 250100, China; 2 Engineering Research Center of Intelligent Unmanned System of Ministry of Education, Shandong University, Jinan 250100, China; 3 School of Mechanical Engineering, Shandong University, Jinan 250100, China; 4 Key Laboratory of High-Efficiency and Clean Mechanical Manufacture of Ministry of Education, Shandong University, Jinan 250100, China; 5 Beijing Advanced Innovation Center for Intelligent Robots and Systems, Beijing Institute of Technology, Beijing 100811, China

**Keywords:** surgical robotics, transluminal endoscopic, surgery, primary–secondary control, singularity, trajectory smooth

## Abstract

This paper presents a miniature manipulator under Cartesian control for minimally invasive transluminal endoscopic surgery. The manipulator had four degrees of freedom (DoFs) and a diameter of only 3.5 mm. The compact size of the manipulator allowed it to pass through the instrument channel of the endoscope, and its high dexterity allowed it to perform most of the operations in endoscopic surgery such as marking, grasping, hanging, etc. The implicit function relationship in the kinematics of the continuum manipulator was analyzed. By introducing the regression analysis method, the analytical form of the inverse kinematics was obtained. The distribution of singularities in the manipulator workspace was analyzed with emphasis. The presence of singularities made Cartesian mapping control between the primary side and secondary side impossible. By introducing the smoothing method of the joint trajectory, the discontinuity of the joint velocity at the singularity was avoided and the primary–secondary mapping under Cartesian control was realized. The trajectory-tracking experiment proved that the smoothness of the joint trajectory could make the manipulator smoothly pass through the singularity. The fixed-point marking experiment proved that the Cartesian control could improve the intuition of operation and the efficiency of task completion. Comprehensive performance experiments showed that the manipulator had enough dexterity to execute complex operations.

## 1. Introduction

Worldwide, the incidence of gastrointestinal cancer among men and women in 2018 ranked fourth and seventh, respectively [1]. There were over one million new cases in 2018 [2]. The 5-year survival rate after treatment for early gastrointestinal tumors can exceed 90%, while the 5-year survival rate for advanced tumors is less than 20% [3]. Therefore, early diagnosis and treatment are the keys to improving survival. Compared with current multi-port laparoscopic surgery and single-port laparoscopic surgery [4,5], minimally invasive endoscopic surgery has the advantages of less trauma, fewer complications, a faster recovery, and a lower cost. It has become the preferred treatment for early gastrointestinal tumors [6].

With the rapid development of surgical robots, minimally invasive transluminal endoscopic surgery robots are receiving an increasing amount of attention. Limited by the characteristics of the digestive tract, traditional rigid surgical instruments cannot meet clinical needs. Surgical instruments need to have a sufficient dexterity, a small cross-sectional diameter, and sufficient flexibility to pass through the narrow and winding natural cavity of a human body to reach the diseased site [7]. The continuum manipulator is widely used in the development of transluminal endoscopic surgery robots due to its good environmental adaptability, small size, and light weight [8]. However, due to the particularity of the continuum mechanism and size constraints, many challenges arise in the design of the drive mechanism, kinematics modeling, and primary–secondary control.

To realize endoscopic surgery through the natural cavity of the human body, there have been many studies on transluminal endoscopic surgery instruments [9,10,11,12]. The “Coba” developed by USGI Medical attempts to solve the issue of triangulation via the addition of three independent arms added to the shapelock-based shaft of the transport [9]. A drawback to the system is that the instruments are fixed and cannot be replaced during operation. The EndoSAMURAI (Olympus Corporation, Tokyo, Japan) is fitted with two bendable hollow arms, which provides two extra DoFs to operate the passive instruments inserted through them [10,11]. The hollow arms are fixed at the end of the endoscope, which makes it difficult to enter the body cavity. The DDES (Boston Scientific) has two 4 mm lumen for the steerable instruments that are capable of performing six degrees of independent motion [12]. The manipulators are all tendon-driven, which has provided a number of references for later researchers. However, these surgical manipulators use a manual drive mode and thus cannot be called a surgical robot system.

Phee et al. developed the “MASTER” transluminal surgical endoscopic robot [13]. The secondary manipulators of the robot adopt a rigid link structure. The manipulator of the gripper has four DoFs and uses a tendon–sheath system to transmit power. Using MASTER, 15 ex vivo and 5 in vivo animal trials have been performed for endoscopic submucosal dissection (ESD). The manipulators attached to the end of the endoscope make it difficult to enter the human body lumen. The “ViaCath” transluminal surgical endoscopic robot developed by Abbott et al. has two manipulators with six DoFs [14]. The manipulators, which are made from a single piece of nylon, have a diameter of 4.75 mm. The manipulators have a high dexterity, but the diameter of the overtube is too large, making it difficult for them to enter the human body cavity. The other major limitation of the ViaCath system is that the instruments can produce only 0.5 N of lateral force. The “STRAS” system developed by De Donno et al. is based on the Anubis^®^ system by Karl Storz [15]. The manipulator of STRAS has three independent DoFs: deflection, rotation, and translation; these can be changed during operation. C. Li et al. developed a miniature manipulator with a variable stiffness for minimally invasive transluminal endoscopic surgery [16]. Each joint of the manipulator is independently controlled by the tendon–sheath system. The stiffness of the manipulator can be adjusted by changing the antagonistic pull of the drive wire during operation. The “K-FlEX” system designed by Hwang et al. has two strong manipulators [17]. The manipulators of “K-FlEX” have five DoFs and can provide a load capacity of 300 g. The high flexibility and compact size of the manipulators allow the system to perform many surgical operations. Although “STRAS” and “K-FlEX” can perform many surgical operations, they still use pseudo-Cartesian control or joint control when performing primary–secondary mapping [18,19]. However, when the primary–secondary mapping adopts pseudo-Cartesian control or joint control, it is demanding for the user, who must mentally construct the elementary displacements required to obtain the desired operational motion.

In this paper, a manipulator that is potentially applicable in minimally invasive transluminal endoscopic surgery is presented. The transmission system can be quickly disassembled from the driver system. This design separates the motor from the transmission system, which not only contributes to the replacement of instruments during the operation but also reduces the difficulty of instrument disinfection. The contributions of this paper can be summarized as follows:

⮚ A miniature manipulator with a diameter of 3.5 mm was proposed that can pass through the instrument channel of the endoscope. The manipulator, which can achieve four DoFs of movement, is driven by a tendon–sheath system and transmission tube. The sufficient dexterity and compact size of the manipulator make it suitable for minimally invasive transluminal endoscopic surgery. The special hollow design of the manipulator allows the forceps sheath to pass through. The forceps sheath, which has a sufficient elasticity, not only acts as the backbone of the manipulator but also decouples the movement of the forceps and manipulator.
⮚ By introducing the regression analysis method, the implicit equation was solved, and the analytical solution of inverse kinematics was obtained. Through geometric analysis of the adjacent joints, the inverse kinematics from the joint space to the drive space was obtained. By comparing the workspace with the task space, the workspace of the manipulator was able to meet the needs of the surgery.
⮚ The singularity and primary–secondary control mode of the manipulator were analyzed. The application of joint trajectory smoothing overcame the speed mutation of the manipulator at the singularity and also realized the Cartesian control of the manipulator. The effectiveness of the trajectory-smoothing algorithm was verified by the trajectory tracking and primary–secondary control experiments. The manipulator’s performance in the dexterous operation procedures was demonstrated in the needle-threading test.

The rest of this paper is organized as follows. In Section 2, the manipulator and drive mechanism are introduced in detail. Section 3 presents the singularities and primary–secondary control. Performance tests are introduced in Section 4, and the conclusions are summarized in Section 5.

## 2. System Description

### 2.1. System Overview

**Figure 1:**
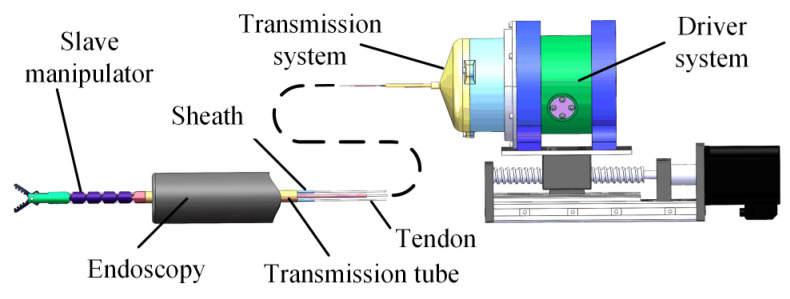
Robotic system.

As shown in Figure 1, the robotics system was composed of three parts: the driver system, the transmission system, and the manipulator. The transmission system and the driver system could be quickly disassembled and assembled, which made the replacement of surgical instruments simple. The detachable transmission mechanism also facilitated the disinfection of surgical instruments. The manipulator and transmission systems were linked by the slender transmission tube. The manipulator could reach the lesion through the instrument channel of the endoscope.

### 2.2. Manipulator

**Figure 2:**
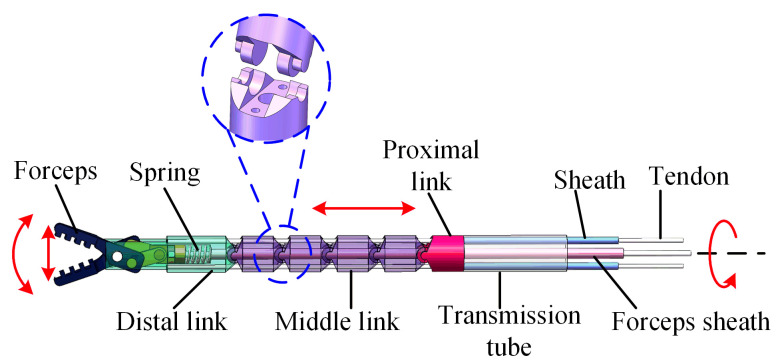
The DoFs and mechanism structure of the manipulator.

As shown in Figure 2, the secondary manipulator consisted of forceps, a distal link, a middle link, a proximal link, and a transmission tube. The manipulator was 26.8 mm in length and 3.5 mm in diameter, which was compact enough to pass through the instrument channel of the endoscope [20]. The manipulator had four DoFs: the translation, rotation, and bending of the manipulator; and the motion of the forceps. The links were in contact with each other through the arc surface, which could rotate freely. To prevent lateral sliding between the adjacent joints, the hinged lugs were designed with a stepped shape. The bending motion of the manipulator was driven by two tendon–sheath systems. The holes (diameter: 0.4 mm) in the link’s sidewall were fabricated via laser drilling, which provided channels for the tendon to pass through. The tendon for the bending motion passed through the proximal link and the middle link in turn and was fixed to the distal link. The sheaths were connected with the proximal link. The motion of the forceps was driven by the tendon–sheath system and spring. The pulling of the tendon closed the forceps. The reaction force of the spring (diameter: 1.8 mm) opened the forceps. The hollow links allowed the forceps sheath (diameter: 0.4 mm) to pass through. The forceps sheath was connected with the distal link. The forceps sheath served as the backbone of the manipulator to improve the rigidity of the manipulator. At the same time, the tendon–sheath system decoupled the motion of the forceps and the bending of the manipulator. The translation and rotation of the manipulator were driven by the transmission tube.

### 2.3. Transmission System

**Figure 3:**
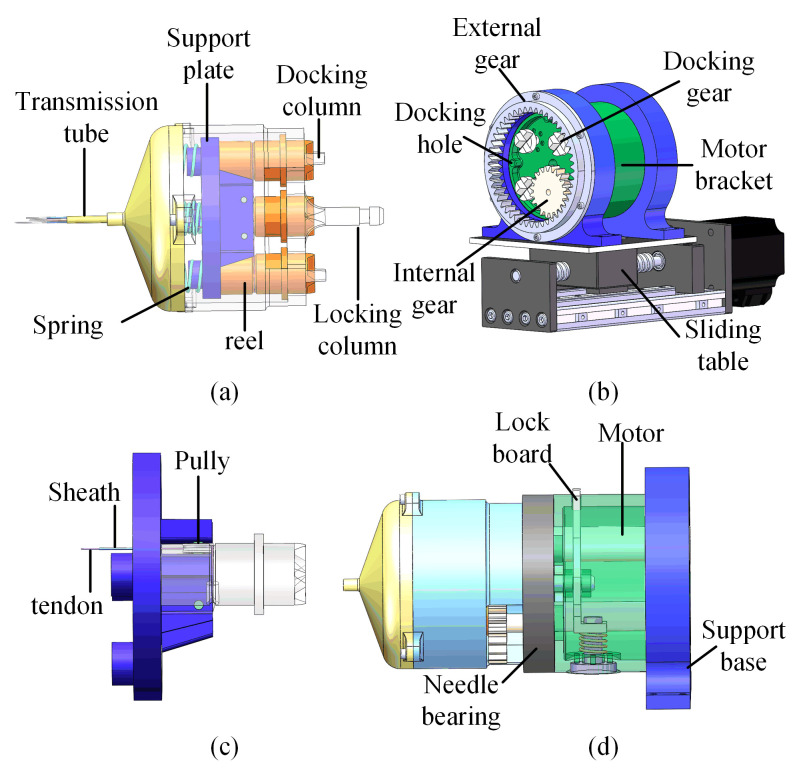
The driver system and transmission system. (**a**) Transmission system of the manipulator; (**b**) driver system of the manipulator; (**c**) close-up view of one tendon and reel configuration; (**d**) close-up view of the detachable mechanism.

As shown in Figure 3a, the transmission tube and tendon–sheath system were connected to the transmission system. The transmission tube was connected to the shell of the transmission system. The tendons were wound on the reel. As shown in Figure 3c, the tendon and sheath were separated by the support plate. The direction of the tendon was changed by the pulley. The plate was supported by four compressed springs as shown in Figure 3a. When the transmission system was disassembled from the driver system, the reel could be pressed on the shell of the transmission system by the support plate, while the pretension of the tendon could not be relaxed. The top of the reels was designed with teeth for easy docking. When the transmission system was assembled with the driver system, the docking gears were connected with the reel. The reels were separated from the shell with the press of the docking gear and could rotate freely. The docking gears were fixedly connected to the motor shaft. The driving force could be transmitted from the motor to the reel. There were three docking columns on the transmission system and three docking holes on the driver system. The docking columns and the docking holes could guarantee the correctness of the docking and the transmission of rotational torque.

### 2.4. Driver System

As shown in Figure 3b,d, three motors with docking gears (RE16, Maxon motor Inc., Sachseln, Switzerland.) were used to drive the tendon–sheath system for manipulator bending and forceps motion. The planetary gear mechanism and a motor (RE19, Maxon motor Inc.) were used to drive the rotation of the manipulator. As shown in Figure 3d, to enable the driver system to rotate freely, needle bearings were used to connect the support base and motor bracket. The planetary gear mechanism could drive the transmission system to rotate, and the transmission system could transmit the rotation to the manipulator via the transmission tube. The quick-disassembly mechanism is shown in Figure 3d. When the locking column was inserted into the center hole of the driver system, the transmission system could be locked by the locking board. When the locking board was pressed, the transmission system could be ejected by the spring.

## 3. Analysis

### 3.1. Kinematics of Manipulator

**Figure 4:**
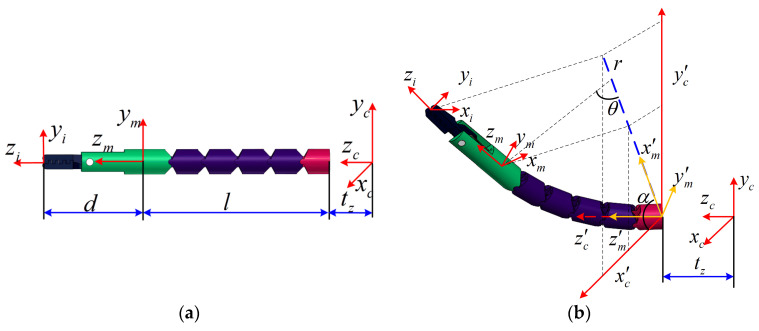
(**a**) Kinematics analysis of manipulator when its bending angle was zero; (**b**) kinematics analysis of manipulator when its bending angle was not zero.

Since the forceps sheath acted as the backbone of the manipulator, the bending manipulator could be approximated as a curve of constant curvature. As shown in Figure 4b, the coordinate system *x_c_y_c_z_c_* was built at the end of the endoscope’s working channel; the *y_c_*-axis was vertical and the *z_c_*-axis coincided with the central axis of the endoscope’s working channel. The direction of the *x_c_*-axis was determined by the right-hand rule. The coordinate system ${x^{\prime }}_{c}{y^{\prime }}_{c}{z^{\prime }}_{c}$ was obtained by moving the coordinate system *x_c_y_c_z_c_* along the *z_c_*-axis for a distance of *t_z_*. The coordinate system ${x^{\prime }}_{m}{y^{\prime }}_{m}{z^{\prime }}_{m}$ was obtained by rotating the coordinate system ${x^{\prime }}_{c}{y^{\prime }}_{c}{z^{\prime }}_{c}$ around the ${z^{\prime }}_{c}$-axis by angle *α*. The coordinate system *x_m_y_m_z_m_* was built at the end of the manipulator, and *x_i_y_i_z_i_* was built at the end of the instrument. The *y_m_*-axis always pointed to the bending drive tendon, and the *z_m_*-axis coincided with the central axis of the end link. We assumed that the bending angle of the manipulator was *θ*, and the rotation angle around the *z_c_*-axis was *α*. As shown in Figure 4b, the conversion process from *x_i_y_i_z_i_* to *x_c_y_c_z_c_* could be divided into three steps [21].Tm′c denotes the transformation matrix from coordinate system ${x^{\prime }}_{m}{y^{\prime }}_{m}{z^{\prime }}_{m}$ to *x_c_y_c_z_c_*,Tmm′ denotes the transformation matrix from coordinate system *x_m_y_m_z_m_* to ${x^{\prime }}_{m}{y^{\prime }}_{m}{z^{\prime }}_{m}$, and Tim denotes the transformation matrix from coordinate system *x_i_y_i_z_i_* to *x_m_y_m_z_m_*. The transformation matrix from coordinate system *x_i_y_i_z_i_* to *x_c_y_c_z_c_* is given in Equation (1).

(1) Tic=Tm′cTmm′Tim=[RicPic01]Tm′c=[cα−sα00sαcα00001tz0001]Tmm′=[cθ0sθl(1−cθ)/θ0100−sθ0cθlsθ/θ0001]Tim=[10000100001d0000]

The posture transformation matrix is given in Equation (2):

(2) Ric=[cθcα−sαcαsθsαcθcαsαsθ−sθ0cθ]

where *cθ = cos*(*θ*), *sθ = sin*(*θ*), *sα = sin*(*α*), and *cα = cos*(*α*). The position of the instrument in the coordinate system *x_c_y_c_z_c_* is expressed in Equation (3) when *θ ≠* 0:

(3) $$P_{i}^{c}=\left[\begin{array}{c} dc\alpha s\theta +lc\alpha (1-c\theta )/\theta \\ ds\alpha s\theta +ls\alpha (1-c\theta )/\theta \\ t_{z}+dc\theta +ls\theta /\theta \end{array}\right]=\left[\begin{array}{c} x_{i}\\ y_{i}\\ z_{i} \end{array}\right]$$

The state of the manipulator when its bending angle was zero is shown in Figure 4a. The position of the instrument is expressed in Equation (4) when *θ* = 0:

(4) $$P_{i}^{c}=\left[\begin{array}{c} 0\\ 0\\ t_{z}+d+l \end{array}\right]=\left[\begin{array}{c} x_{i}\\ y_{i}\\ z_{i} \end{array}\right]$$

where *t_z_* denotes the translation distance of the secondary manipulator, *θ* is the bending angle, *α* is the rotation angle, *l* is the length of the bending section, and *d* is the length of the rigid part at the instrument.

By comparing Equations (2) and (3) when the bending angle *θ* ≠ 0, the orientation and position of the instrument are given by the bending angle and the rotation angle. In this case, if the position of the instrument is given, the orientation can also be determined, the orientation and the position are coupled. Compared with orientation, position control is more meaningful for operation. By comparing Equations (3) and (4), when *θ* = 0, the position of the instrument is independent of rotation angle *α*, and the position can only be adjusted by the translational DoF *t_z_*. In this case, the DoF of *α* was lost. This meant that *θ* = 0 was a singularity within the workspace.

### 3.2. Inverse Kinematics

To implement the primary–secondary control of the manipulator, the inverse kinematics model had to be established. The joint variable *q_i_* (*θ*, *α*, *t_z_*) had to be expressed by the position variable $P_{i}^{c}$(*x_i_*, *y_i_*, *z_i_*). According to Equation (3), we could easily obtain the rotation angle using Equation (5):

(5) $$\alpha =\mathrm{atan}2(y_{i},x_{i})$$

When the rotation angle *α* was solved, the bending angle *θ* could be solved using Equation (6) or Equation (7):

(6) $$x_{i}=dc\alpha s\theta +lc\alpha (1-c\theta )/\theta$$

or

(7) $$y_{i}=ds\alpha s\theta +ls\alpha (1-c\theta )/\theta$$

Since Equations (6) and (7) are in the form of a transcending equation, the analytical solution of *θ* could not be obtained directly.

Actually, there was a hidden condition that could be applied in Equation (3). It can be seen in Equation (3) that when the translational DoF *t_z_* is not considered, the distance between the tip of the instrument and the coordinate planes ${x^{\prime }}_{c}{o^{\prime }}_{c}{y^{\prime }}_{c}$ can be defined as Equation (8):

(8) $$z_{i}{}^{\prime }=dc\theta +ls\theta /\theta$$

As shown by the blue dotted line in Figure 4b, *r* denotes the distance between the tip of the instrument and the ${z^{\prime }}_{c}$-axis. The distance *r* can be expressed as Equation (9):

(9) $$r^{2}=x_{i}^{2}+y_{i}^{2}$$

According to Equations (6) and (7), Equation (9) can be rewritten as Equation (10):

(10) $$r^{2}={\left[ds\theta +l(1-c\theta )/\theta \right]}^{2}$$

**Figure 5:**
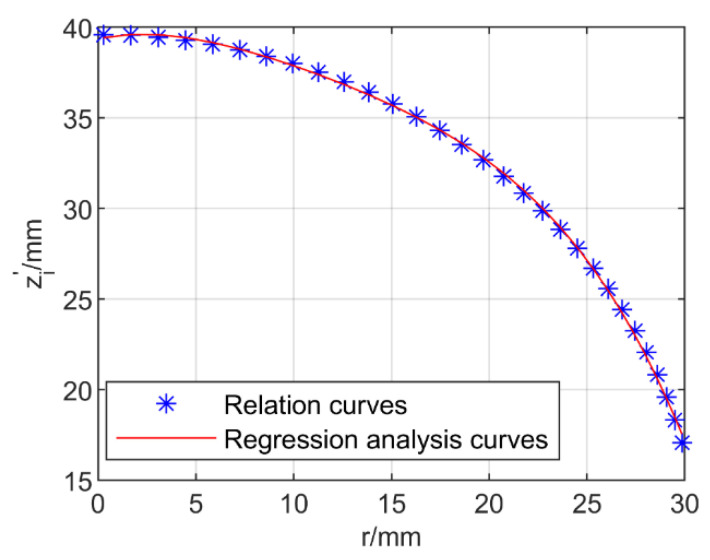
Regression analysis of the relation curves.

It can be seen in Equations (8) and (10) that there is a hidden relationship between *r* and ${z^{\prime }}_{i}$. In this paper, the length of the rigid instrument was *d* = 12.8 mm, and the length of the flexible part of the manipulator was *l* = 26.8 mm. As shown in Figure 5, by traversing the bending angle *θ*, the relation curves between *r* and ${z^{\prime }}_{i}$ could be obtained.

The relation curves can be represented by a quartic equation. As shown in Figure 5, a new function for *r* and ${z^{\prime }}_{i}$ can be obtained by applying a regression analysis method, which can be expressed by Equation (11):

(11) $${z^{\prime }}_{i}=a_{1}r^{4}+a_{2}r^{3}+a_{3}r^{2}+a_{4}r+a_{5}$$

The coefficients *a*_1_ to *a*_5_ in Equation (11) can be determined via regression analysis. According to Equations (9) and (11), when the coordinate position *x_i_*, *y_i_* is given, the distance ${z^{\prime }}_{i}$ can be obtained. Through Equations (6) and (8), *θ* can be expressed as Equation (12):

(12) $$\theta =2\arctan(\frac{x_{i}}{({z^{\prime }}_{i}+d)c\alpha })$$

Through Equations (7) and (8), *θ* can be expressed as Equation (13):

(13) $$\theta =2\arctan(\frac{({z^{\prime }}_{i}+d)s\alpha }{y_{i}})$$

When the bending angle *θ* is solved, the translation distance can be easily expressed by Equation (14). So far, the inverse kinematics from the workspace to the joint space have been obtained. Refer to our previous work [22,23] for other methods to find the inverse kinematics of the continuum manipulator.

(14) $$t_{z}=z_{i}-ls\theta /\theta -dc\theta$$

**Figure 6:**
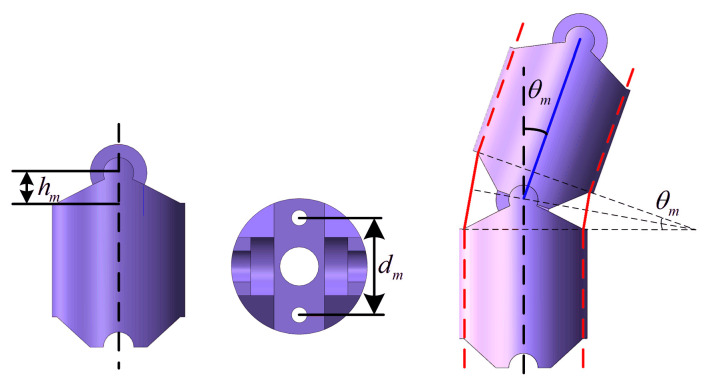
Geometrical analysis of the joints.

As shown in Figure 6, *d_m_* denotes the distance between the cable holes, *θ_m_* denotes the rotation angle between the adjacent joints, *n* is the number of manipulator joints, and *h_m_* is the height of the joint shaft. The variation in length of the action tendon in the pulling direction and releasing direction can be presented as Δ*l_p_* and Δ*l_r_*, respectively. The length variation in the pulling direction was defined as positive. According to the geometrical analysis of the adjacent joints, Δ*l_p_* and Δ*l_r_* can be expressed as Equation (15):

(15) $$\{\begin{array}{c} \Delta l_{p}=n(2h_{m}(1-\cos\frac{\theta_{m}}{2})+d_{m}s\frac{\theta_{m}}{2})\\ \Delta l_{r}=n(2h_{m}(1-\cos\frac{\theta_{m}}{2})-d_{m}s\frac{\theta_{m}}{2}) \end{array}$$

The relationship between the bending angle *θ* and joint angle *θ_m_* can be expressed by Equation (16):

(16) $$\theta_{m}=\frac{\theta }{n}$$

So far, the inverse kinematics from the joint space to the drive space have been obtained.

### 3.3. Jacobian and Singularities

To analyze the relationship between the linear velocity and joint velocities of the manipulator, the Jacobian could be obtained by taking the partial derivative of the position transformation matrix Pci. The Jacobian (P˙ci=Jq˙i) is expressed in Equation (17) when *θ* ≠ 0:

(17) $$J=\left[\begin{array}{ccc} \frac{\partial x_{i}}{\partial \theta } & -y_{i} & 0\\ \frac{\partial y_{i}}{\partial \theta } & x_{i} & 0\\ \frac{\partial z_{i}}{\partial \theta } & 0 & 1 \end{array}\right]\;\mathrm{for}\;\theta \neq \;0$$

When the bending angle of the manipulator was zero as shown in Figure 4a, the Jacobian could be obtained by taking the limit of the joint angle at *θ* = 0 using Equation (3). Then the Jacobian was rewritten as Equation (18):

(18) $$J=\left[\begin{array}{ccc} c\alpha (d+l/2) & 0 & 0\\ s\alpha (d+l/2) & 0 & 0\\ 0 & 0 & 1 \end{array}\right]\;\mathrm{for}\;\theta =0$$

As shown in Equation (18), when *θ* = 0, the determinant of ***J*** is null and the manipulator is in a singular posture. Consequently, one of the DoFs is lost. The rotation angle *α* has no effect on changing the instrument position. The position of the manipulator can only be changed by the translation movement *t*_z_.

**Figure 7:**
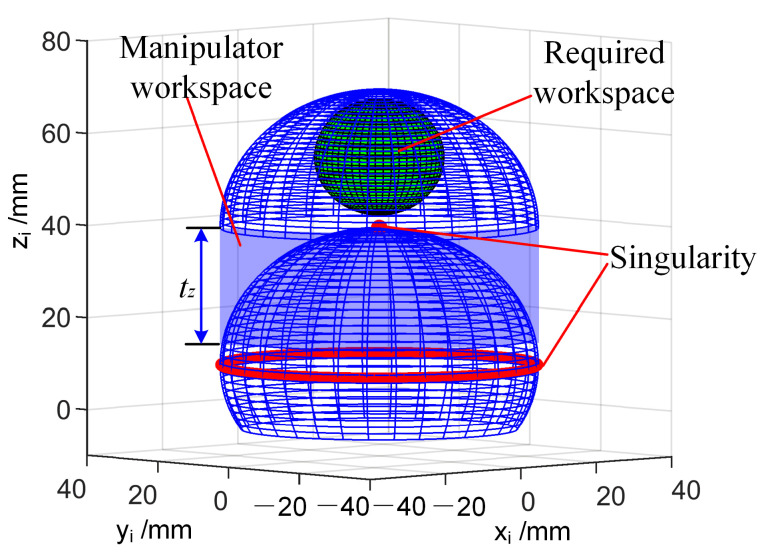
The singularity and workspace of the manipulator.

According to the analysis of Equation (17), when *θ* ≠ 0, the determinant of ***J*** is expressed in Equation (19). When the determinant of ***J*** is null, the other singularities of the manipulator can be obtained at *θ* = 107.2° as shown by the red circle in Figure 7. As shown in Figure 7, the blue part represents the workspace of the manipulator with translation DoF, and the red mark is the singularity of the manipulator. The required workspace for ESD is a spherical area with a diameter of 25 mm [20]. In Figure 7, the green spherical portion represents the required workspace, so the workspace of the manipulator could meet the needs of transluminal endoscopic surgery.

(19) $$\Delta =\frac{\partial x_{i}}{\partial \theta }x_{i}+\frac{\partial y_{i}}{\partial \theta }y_{i}=\frac{\partial (x_{i}^{2}+y_{i}^{2})}{\partial \theta }$$

When the continuum manipulators performed a coordinated operation, the best range of intersection angle of the two manipulators was [90°, 180°] [24]. The bending angle of the manipulator had to be less than 90°, so the singularity at *θ* = 107.2° did not need to be considered. As shown in Figure 7, the singularity at *θ* = 0 was just in the vertex of the workspace, and the joint velocity was discontinuous at the singularity. So, the singularity at *θ* = 0 had to be dealt with. This will be discussed in detail in next section.

### 3.4. Singularity Transition

**Figure 8:**
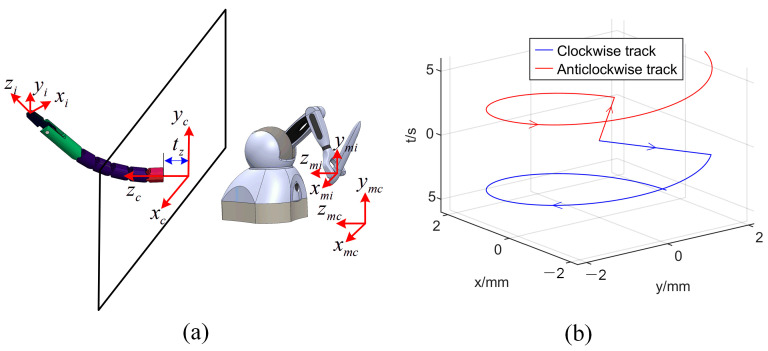
(**a**) Mapping relationship between the haptic device and manipulator; (**b**) two reference trajectories of the manipulator starting from the origin.

**Figure 9:**
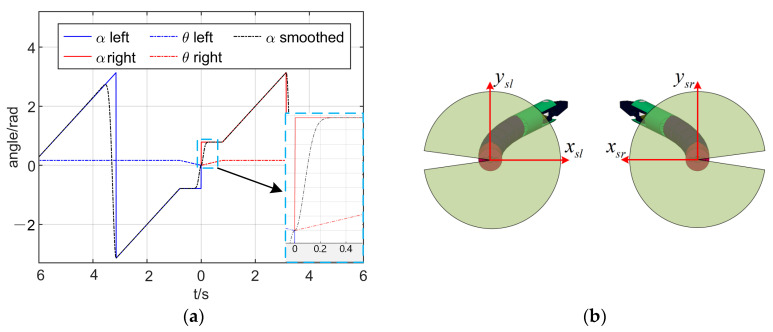
(**a**) Original joint trajectory and smoothed joint trajectory; (**b**) schematic diagram in two-manipulator collaboration mode. The green area indicates the range of the rotation angle.

The Cartesian mapping relationship between the haptic device and manipulator is shown in Figure 8a, in which $x_{mc}y_{mc}z_{mc}$ is the origin coordinate system of the relative motion of the haptic device, and $x_{mi}y_{mi}z_{mi}$ is the coordinate system fixed on the haptic device. To facilitate the preloading of the drive tendon and the correct mapping of the Jacobian matrix, the manipulator had to start from the initial position. The singularity at *θ* = 0 could not be avoided. Through the previous analysis, it was seen that the singularity mainly affected the rotation angle *α* with no effect on translation *t_z_*. To analyze the effect of the singularity on the joint velocity, the track of the manipulator under the control of the haptic device was assumed as shown in Figure 8b. The red and blue lines indicate two trajectories starting from the origin. The manipulator first made a linear movement along the angles of *π*/4 and −*π*/4 and then made a circular movement with a fixed radius in the opposite direction. According to the inverse kinematics of the manipulator analyzed in the previous section, the joint trajectory curve corresponding to the trajectory of the manipulator could be solved as shown in Figure 9a.

**Figure 10:**
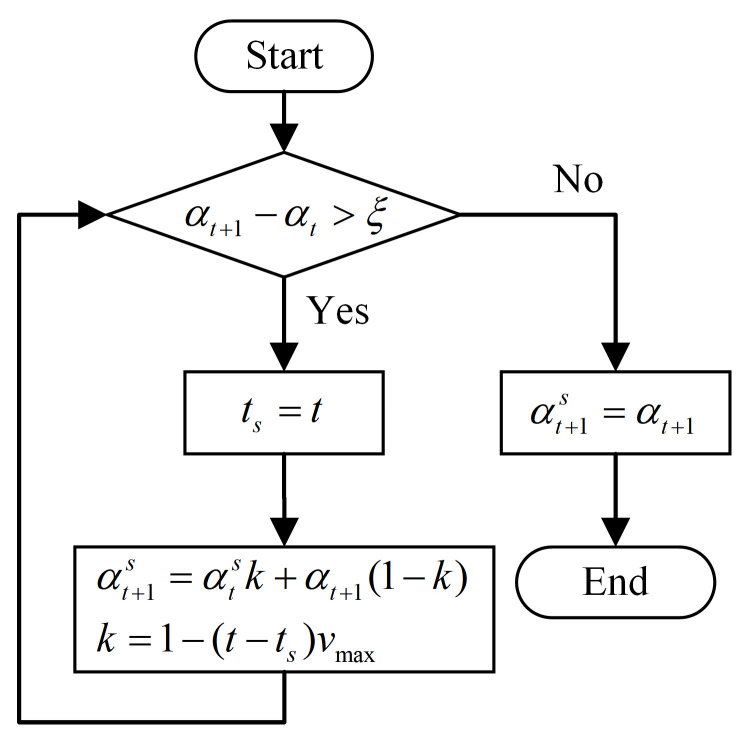
Flow chart of trajectory-smoothing process.

In Figure 9a, it can be seen that the trajectory of the bending angle *θ* started from 0 and was continuous. The singularity had no effect on the trajectory of the bending angle. The trajectory of the rotation angle *α* was discontinuous at the singularity. The jump of this trajectory caused the vibration of the motor and even the failure of the motor driver. Using pseudo-Cartesian control or joint control for primary–secondary mapping can solve the problem of discontinuous joint trajectories [18] but will cause the operator to lack intuition. The operator must mentally construct the elementary displacements required to obtain the desired operational motion. To realize the Cartesian control, the discontinuity at the singularity must be smoothed. In this paper, Equation (20) was used to smooth the trajectory of the rotation angle at the singularity:

(20) $$\{\begin{array}{l} \alpha_{t+1}^{s}=\alpha_{t}^{s}k+\alpha_{t+1}(1-k)\\ k=1-(t-t_{s})v_{\max},k>0 \end{array}$$

where $\alpha_{t+1}^{}$ denotes the original angle of the next moment, $\alpha_{t+1}^{s}$ is the smoothed angle of the next moment, *k* is the smoothing scale factor, *v_max_* is smoothing speed to be determined by the maximum acceleration of the system, and *t_s_* is the start time of the smoothing operation to be judged by comparing the rotation angle of the current moment and the rotation angle of next moment. The trajectory-smoothing process is shown in Figure 10, in which *ξ* represents the threshold used to judge the abrupt change of the trajectory.

The smoothened trajectory of the rotation angle is shown as the black dashed line in Figure 9a. The smoothened trajectory replaced the original trajectory at the singularity, which restored the original trajectory tracking in a short time. By smoothing the trajectory of the rotation angle at the singularity, Cartesian control could be realized.

It can be also seen in Figure 9a that the trajectory of the rotation angle had abrupt changes at −*π* and *π*, which also caused the motor driver to malfunction. The abrupt changes at −π and π can be explained by Equation (5). To prevent the motor driver from malfunctioning, we restricted the rotation angle range of the manipulator. In Figure 9b, the green transparent area represents the range of the rotation angle of the manipulator. *x_sl_y_sl_z_sl_* and *x_sr_y_sr_z_sr_* denote the left-manipulator and right-manipulaotr coordinate systems established at the end of the endoscopic channel, respectively; the *x_sl_y_sl_z_sl_* coordinate system followed the left-hand rule and the *x_sr_y_sr_z_sr_* coordinate system followed the right-hand rule. The z*_sr_*-axis and z*_sl_*-axis are pointing inward perpendicular to the paper in the figure. The manipulator of the transluminal endoscopic surgery robot mostly worked in a dual-manipulator collaborative mode [10,11,12,13,14,15] and did not need to operate the part outside the limited area. At the same time, the part outside the constrained area was in the blind zone of the endoscope’s visual field. The restricted range of rotation angle had no effect on the actual operation.

## 4. Experiments and Results

The power transmission between the transmission system and the manipulator was carried out by the tendon–sheath system. The tendon would elastically elongate due to the friction and tension; the elongation was related to the total bending angle of the sheath. To ensure the movement accuracy of the manipulator, we compensated the elastic elongation of the cable [25].

### 4.1. Trajectory-Tracking Experiment

**Figure 11:**
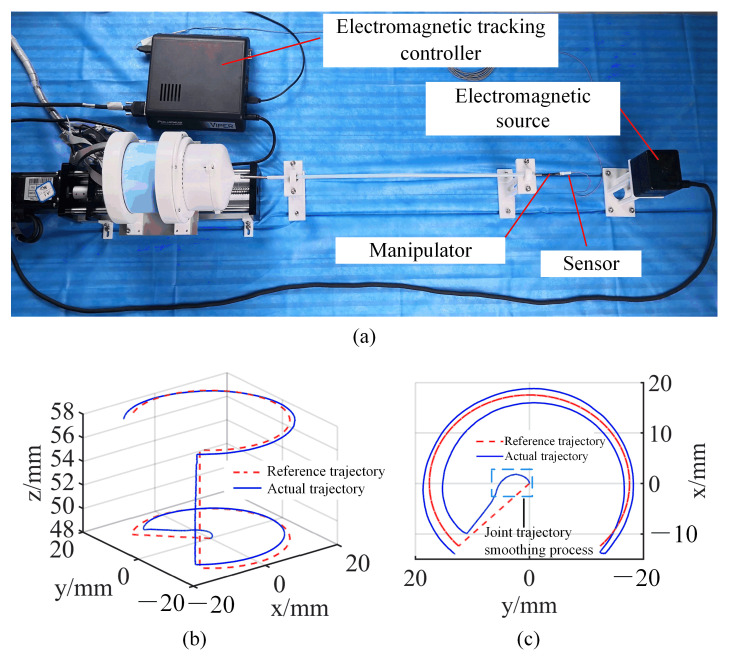
(**a**) Experimental setup of trajectory tracking; (**b**) isometric view of trajectory tracking; (**c**) top view of trajectory tracking.

In this section, the performance of manipulator trajectory tracking is evaluated. An experimental setup was built to verify the trajectory-tracking performance when the manipulator was operated in a Cartesian control mode. The experimental setup is shown in Figure 11a. The motors of the driver system were driven by the digital controller (G-SOLTWI6/100EE01 from Elmo Group), and the digital controller was connected to an industrial computer (C6920 from Beckhoff Group) by the Ethercat (Controller Area Network) bus. A teflon tube with an inner diameter of 4 mm was fixed on the test bench by a bracket. The manipulator could be freely bent and rotated through the Teflon tube. An electromagnetic tracking system (resolution: 0.001 mm; VIPER 4, Polhemus Ltd., Vermont, USA) was used to measure the trajectory of the manipulator. The electromagnetic (EM) sensor (diameter: 1.8 mm) was mounted on the end of the manipulator. The forceps were replaced with a 3D-printed bracket for easy mounting of the EM sensor. An electromagnetic source was placed in the front of the manipulator to ensure that the sensor was within the detection range. The electromagnetic tracking controller was connected to the industrial computer with a USB cable.

The motion trajectory of the manipulator is shown in Figure 11b,c. As shown in Figure 11b, the motion trajectory of the manipulator could follow the reference trajectory. The motion of the manipulator could start from the origin under Cartesian control, and the smoothing algorithm of joint trajectory solved the problem of the motor failing at the singularity. The arc at the center of Figure 11c presents the smoothing process of joint trajectory. The manipulator tracked the reference trajectory at the origin by interpolating the motion. As can be seen in Figure 11c, although the necessary compensation was made for the elongation of the drive wire, because the manipulator moved under open-loop control, there was an error between the motion trajectory and the reference trajectory. This error could be further reduced by the operator when the manipulator operated in hand–eye coordination mode.

### 4.2. Test for Primary–Secondary Control

**Figure 12:**
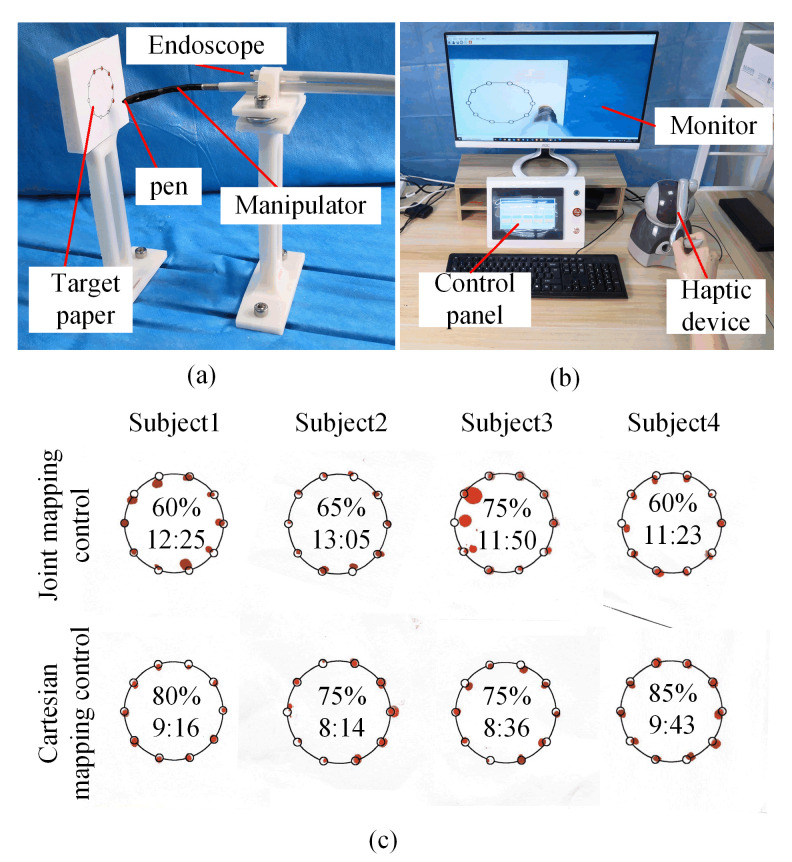
Test for master–slave control: (**a**) Slave side of experiment setup for master–slave control test; (**b**) Master side of experiment setup for primary–secondary control test; (**c**). Experiment result of master–slave control.

This experiment was conducted to mark the fixed point in the workspace. As shown in Figure 12a, a Teflon tube with both ends fixed was used to simulate the instrument channel of the endoscope, and a micro camera (diameter: 3.9 mm, resolution: 1280 × 720) that was fixed above the manipulator was used to capture the operational images. The forceps of the manipulator were replaced with a pen. The graph paper was placed on the left front of the manipulator. As shown in Figure 12b, a haptic device was used on the primary side. A monitor was placed in front of the operator for operation observation. The control panel was used for parameter adjustment. The primary side and secondary side were mapped using Cartesian control. Four subjects were invited to control the manipulator to target every small circle laid on the large circle. The small and large circles were 2 and 25 mm in diameter, respectively. This experiment simulated the procedure of marking a lesion during an operation [26].

Figure 12c shows the accuracy and time required to complete the task when the manipulator was operated in Cartesian mapping control and joint mapping control, respectively. The pen was almost tangent to the graph paper when the manipulator tried to mark the small circle on left of the graph paper, which made the marking extremely difficult. In actual surgery, this problem can be solved by adjusting the position of the endoscope. In this experiment, the graph paper and the base of the manipulator were fixed at the same time, which verified that the workspace of the manipulator could meet the requirements. Due to the intuitive feeling of the Cartesian control, the accuracy and time required for these four subjects to complete the task improved by 11.25% and 3.2 s, respectively.

### 4.3. Performance Test

**Figure 13:**
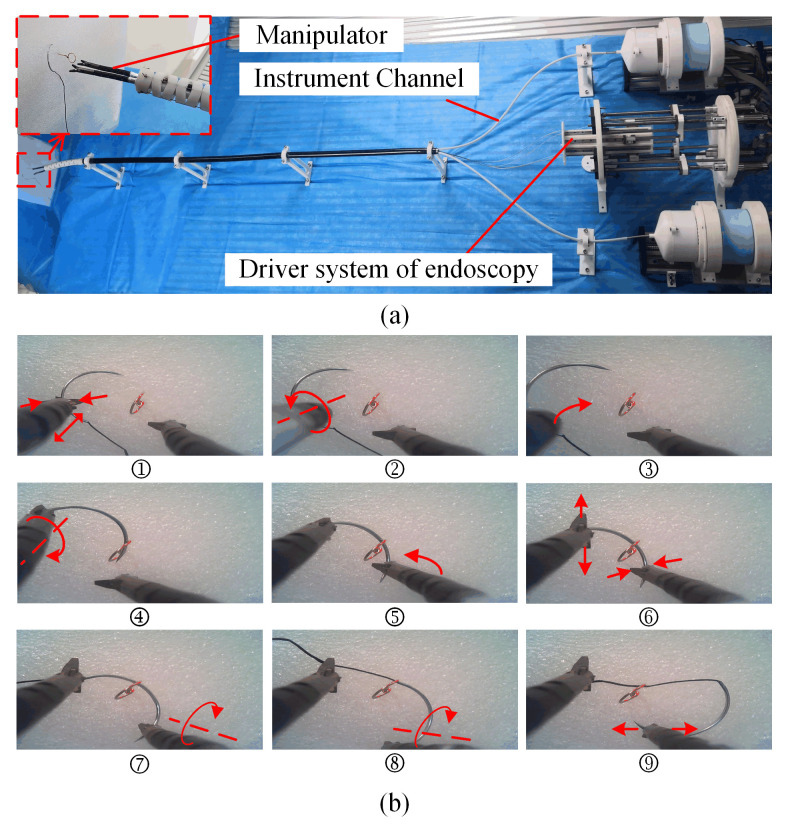
Performance test. (**a**) Experimental setup; (**b**) Operation process.

A possible cause of postoperative bleeding and perforation is that the large mucosal defect after endoscopic surgery remains open [27]. A defective mucosa is eroded by digestive juices and is difficult to heal, which can easily lead to blood vessel rupture or perforation. The application of hemoclips and a snare can achieve the closure of large mucosal defects, but when considering the safety and economic benefits, suturing is still the best choice [28]. Suturing with stitches and knots, which is widely adopted in open or laparoscopic surgery, is still rarely used in endoscopic surgery [29]. To test the flexibility and collaborative ability of the manipulator, we carried out a needle-threading experiment. The secondary side of the experimental setup is presented in Figure 13a, including two manipulators and driver systems as well as a simulated endoscope platform (outer diameter: 14 mm) and its driver system.

The simulated endoscope was fixed on the test bench. The flexible part of the endoscope was fixed to simulate that the endoscope was supported by the tissue. There were two instrument channels in the simulated endoscope platform that allowed the manipulators to pass through. A micro camera that was used in the last experiment was mounted on the end of the simulated endoscope. The primary side of the experimental setup was the same as that shown Figure 12b except that there were two haptic devices placed in front of the operator. A foot switch was used to switch control between the manipulator and the simulated endoscope. The plastic foam board was placed in front of the simulated endoscope platform. A metal ring (diameter: 4 mm) was fixed on the foam board. The needle with thread (30 mm, 1/2 round bodied) for surgical suturing was mounted near the ring before the experiment began.

The operation procedure is shown in Figure 13b. The left manipulator transmitted to the suture needle. The forceps began to close as they approached the needle (Figure 13b-①). The suture needle was removed from the foam board using a counterclockwise rotation of the manipulator (Figure 13b-②). The manipulator bent and rotated to bring the suture needle close to the metal ring (Figure 13b-③). When the needle tip was close to the ring, the manipulator mainly rotated to make the suture needle pass through the ring (Figure 13b-④). When the suture needle was halfway through the ring, the right manipulator bent to get close to the suture needle (Figure 13b-⑤). When the right forceps approached the suture needle, the right forceps began to close and the left forceps began to open (Figure 13b-⑥). After the right forceps captured the suture needle, the right manipulator rotated to make the suture needle pass through the metal ring (Figure 13b-⑦). To avoid the interference between the suture needle and the metal ring, the right manipulator had to be accompanied by a bending motion while rotating (Figure 13b-⑧). When the suture needle completely passed through the metal ring, the right clamp was opened and the entire experiment was completed (Figure 13b-⑨).

The experiment, which lasted 3 min and 30 s, verified that the manipulator could complete all degrees of freedom of movements such as translation, rotation, bending, and forceps opening and closing. It also proved that the manipulator had a sufficient dexterity to complete some complex operations. In Cartesian control mode, the operator was more intuitive, which was very beneficial to controlling the position of the end of the manipulator. To the best of our knowledge, similar experiments have not been reported previously in the literature on endoscopic surgical robots.

## 5. Conclusions

This paper proposed a novel miniature manipulator with four DoFs for minimally invasive transluminal endoscopic surgery. The small diameter (3.5 mm) of the manipulator enabled it to pass through the instrument channel of the endoscope. The manipulator consisted of a plurality of discrete cylindrical joints. Two tendons passed through the holes in the sidewall of the joints to drive the manipulator to bend. The driver system and transmission system were designed for quick disassembly and assembly, which enabled different instruments to be quickly replaced during the operation. The kinematics and inverse kinematics of the manipulator were analyzed. Implicit equations were transformed into explicit equations by applying a regression analysis, so a need to solve transcendental equations was avoided and an analytical solution of the inverse kinematics was obtained. The distribution of singularities in the workspace was determined by the analysis of the Jacobian matrix. Through the simulation of the typical trajectory of the manipulator, the influence of the singularities on the joint trajectory was analyzed. By smoothing the joint trajectory, the Cartesian control of the manipulator was realized.

To analyze the effect of joint trajectory smoothing on the trajectory of the manipulator, a reference trajectory-tracking experiment was performed. This experiment showed that the manipulator could pass through the singularity by smoothing the joint trajectory, while the manipulator could quickly follow the reference trajectory by way of motion interpolation. To analyze the effect of the Cartesian control on the manipulator, a fixed-point labeling experiment was carried out. This experiment compared the accuracy and time for task completion in different modes and showed that the Cartesian control could effectively improve the control accuracy and task efficiency. To analyze the flexibility and cooperation of the manipulators, a suture-needle-threading experiment was performed. This experiment showed that the novel manipulator could perform complex operations. The realization of the needle-threading experiment was of great significance with regard to the endoscopic surgery robot’s ability to pass through the stomach wall and enter the abdominal cavity to perform surgery in the future.

In this paper, the basic performance of the manipulator was analyzed and verified. In the future, we will implement experiments using animal organs. More performances will be verified, including the load capacity and the ability of the forceps to grip tissue, tie knots, etc.

## Data Availability

Not applicable.
